# Supplementary material for: Cryo‐EM structure of native human uromodulin, a zona pellucida module polymer
Source: EMBO J. 2020 Nov 16;39(24):e106807. doi: 10.15252/embj.2020106807 (PMC7737619; doi:10.15252/embj.2020106807)
Supplement: Supplementary file 1 — Appendix [file EMBJ-39-e106807-s001.pdf]

Appendix

Cryo-EM structure of native human uromodulin, a zona pellucida module polymer

Alena Stsiapanava†, Chenrui Xu†, Martina Brunati, Sara Zamora-Caballero, Céline Schaeffer, Marcel Bokhove, Ling Han, Hans Hebert, Marta Carroni, Shigeki Yasumasu, Luca Rampoldi, Bin Wu* & Luca Jovine**

*,** Corresponding authors. E-mail: wubin@ntu.edu.sg, luca.jovine@ki.se
† These authors contributed equally to this work

Table of contents

Appendix Figure S1 - page 2

Appendix Figure S2 - page 4

Appendix Figure S3 - page 6

Appendix Table S1 - page 7

Appendix Materials and Methods - page 8

Appendix References - page 10

© 2020 The Authors.

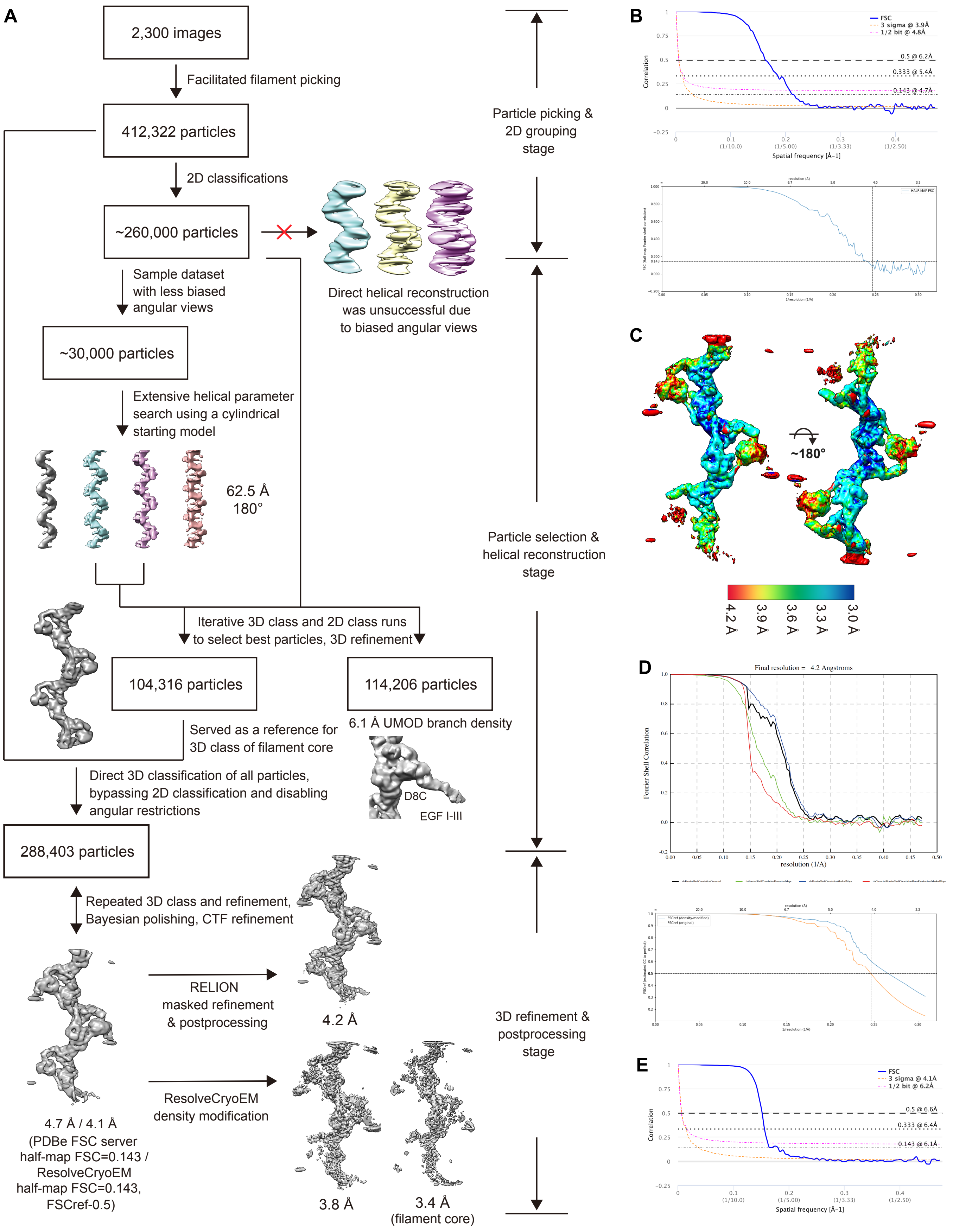

Appendix Figure S1. 3D helical reconstruction of the UMOD filament.

© 2020 The Authors.

A Helical reconstruction of the 3D density map of $UMOD_{fl}$ was performed in three steps. (1) Particle picking and 2D grouping. Filaments were picked with the help of contrast-enhancing programs and pre-filtered based on contrast and clarity of the segments. Once helical pitch and power spectrum were obtained, we attempted direct 3D helical reconstruction, but failed to obtain reasonable density. (2) Searching for the true helical parameters and selecting the best group of particles. After realizing that it was necessary to pool different 2D classes together, for time efficiency we selected a subset of 30,000 particles with the best contrast and better angular distribution and used them to iteratively sample potential combinations of helical parameters consistent with the 2D views. Once the correct helical parameters were determined, we gradually included more particles in the 3D classification and refinement procedures. Since some of the angular views were intrinsically weaker than the others, we bypassed the 2D classification step and proceeded with 3D classification of all the extracted particles. Eventually, we selected 288,403 particles that aligned with a single 3D class density that best depicts the filament core, and 114,206 particles that best illustrate the extended arm density, during this iterative selection and grouping process. (3) 3D refinement and postprocessing. All selected particles were combined and used in a few final rounds of 3D refinement, with gradually reduced sampling steps. This eventually led to a 3D helical reconstruction density of the filament core part of $UMOD_{fl}$ with significantly improved map features and nominal resolution, which was further polished by postprocessing in RELION or density modification using ResolveCryoEM. The RELION multi-body module was then used to perform a focused refinement of the branch density to 6.1 Å, using the selected group of 114,206 particles whose branches aligned well during 3D classification. A final composite map representing the entire $UMOD_{fl}$ molecule was built by combining the separately refined filament core and branch densities.

B Half-map FSC curves for the $UMOD_{fl}$ reconstruction generated by PDBe FSC (top) or ResolveCryoEM (bottom).

C Unsharpened cryoEM map of $UMOD_{fl}$, colored by local resolution as estimated by ResMap.

D Gold-standard FSC plot from RELION postprocessing (top) and ResolveCryoEM FSCref correlation between the initial map or the density-modified map and a true map (bottom).

E Half-map FSC curve, generated by PDBe FSC, for the local map reconstruction of the $UMOD_{fl}$ branch.

© 2020 The Authors.

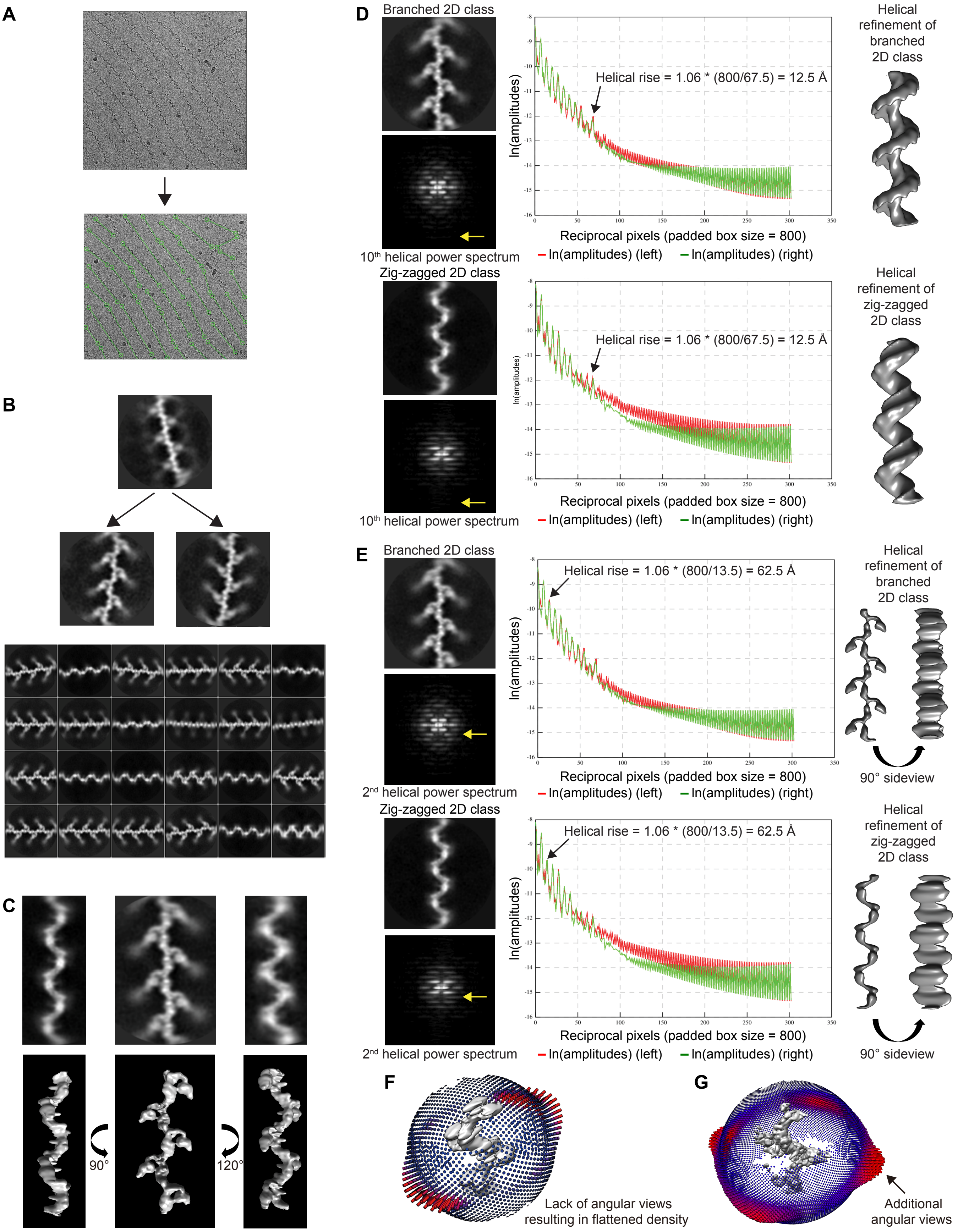

Appendix Figure S2. Initial 2D classification and helical reconstruction of the UMOD filament.

 © 2020 The Authors.

A Auto-picking of UMOD filaments with manual adjustment.

B Example of a symmetric 2D class resulting from averaging of filaments with opposite polarities (top), separation thereof by iterative 2D classification (middle) and extension of the same procedure to other classes to obtain a set of non-symmetric 2D averages (bottom).

C Highly different $UMOD_{fl}$ 2D classes (top) and corresponding angular views of the $UMOD_{fl}$ filament density after 3D classification (bottom).

D Conventional helical reconstruction of individual 2D classes was unsuccessful.

E Re-examination of alternative helical symmetry identified the correct helical rise value. Each 2D class is a single angular view of the UMOD filament, and not sufficient for 3D reconstruction. Thus, it was necessary to pool the different orientations together.

F Biased angular distribution in the initial 3D refinement.

G Inclusion of particles with additional angular views and finer angular sampling helped 3D classification significantly.

© 2020 The Authors.

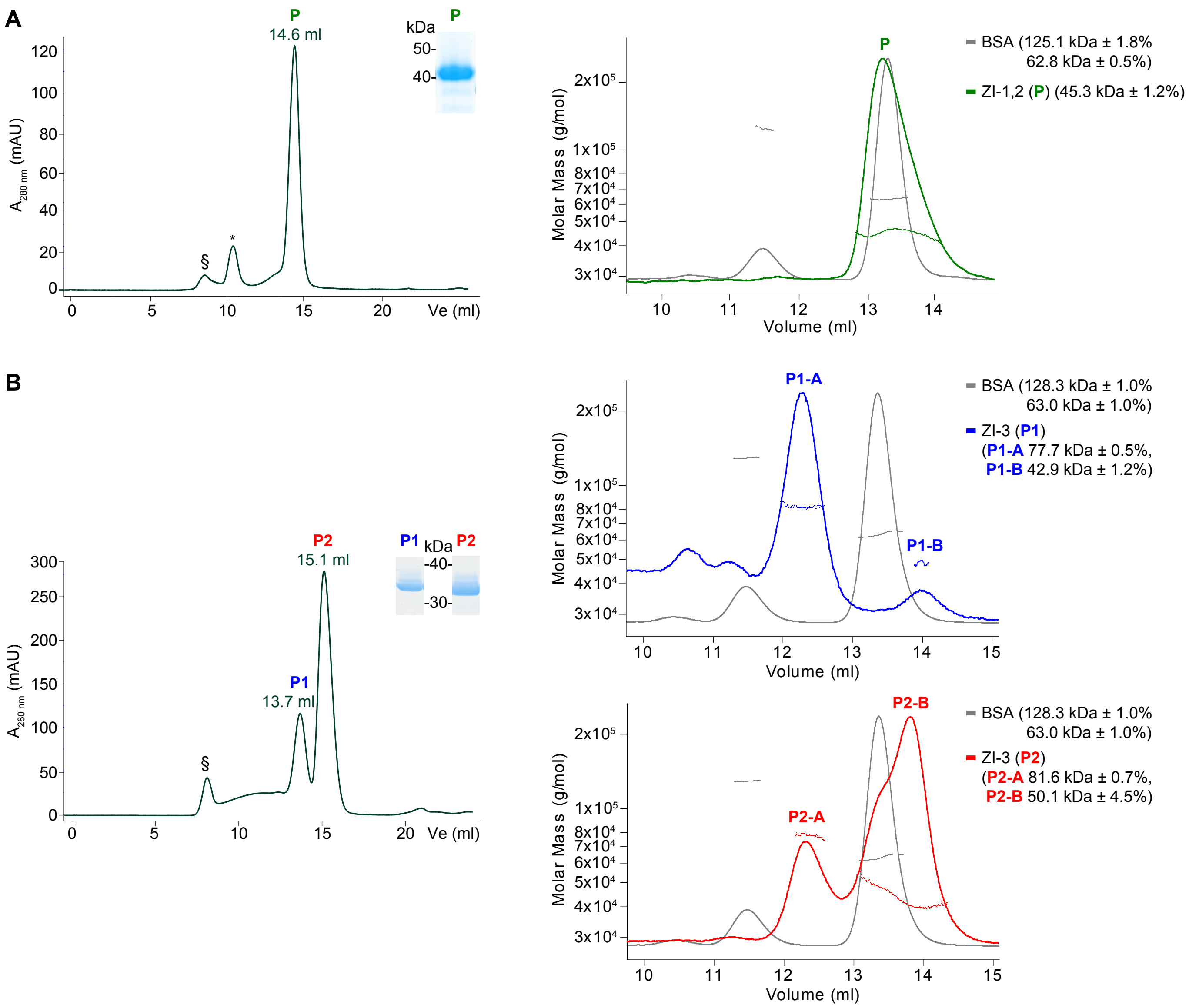

Appendix Figure S3. Analysis of the oligomeric state of fish egg coat proteins.

IMAC-captured His-tagged proteins were purified by SEC (left panels) and relevant elution peaks were analyzed by reducing Coomassie-stained SDS-PAGE (left panel insets) and SEC-MALS (right panels). SEC void volume and contaminant peaks are indicated by § and *, respectively. SEC-MALS experiments were calibrated using bovine serum albumin (BSA). All experiments were repeated independently twice, with similar results; for clarity, a single experiment for each protein is presented in the figure.

A Medaka ZI-1,2 is a monomer in solution.

B Medaka ZI-3 elutes as two SEC peaks that correspond to homodimeric (P1) and monomeric (P2) forms of the glycoprotein.

Source data are available online for this figure.

 © 2020 The Authors.

Appendix Table S1. Cryo-EM data collection, refinement and validation statistics.

	**UMOD$_{fl}$**<br>EMDB: EMD-10553<br>PDB: 6TQK	**UMOD$_{e}$**<br>EMDB: EMD-10554<br>PDB: 6TQL
**Data collection and processing**		
Magnification	130,000	165,000
Voltage (kV)	300	300
Electron exposure (e–/Å^2)	39.6	44.6
Defocus range (μm)	-1.5 – -3.5	-1.4 – -3.0
Pixel size (Å)	1.06	0.82
Symmetry imposed	Helical (62.5 Å rise, 180.0° rotational angle)	Helical (62.7 Å rise, -179.9° rotational angle)
Initial particle images	412,322	252,438
Final particle images	288,403	94,937
Unmasked raw map resolution (Å) (FSC = 0.143)	4.7	6.0
Unmasked raw map resolution range (Å)	3.0 – 5.4	4.2 – 6.6
Postprocessed map resolution (Å)		
ResolveCryoEM (FSCref = 0.5)	3.8 | 3.4 (filament core)	4.0
RELION (FSC = 0.143)	4.2	4.3
**Refinement**		
Initial model used (PDB code)	4WRN	6TQK
Model composition		
Non-hydrogen atoms	4,919	4,890
Protein residues	593	587
Carbohydrate residues	22	24
B factors (Å^2)		
Protein	135	178
Carbohydrate	170	204
R.m.s. deviations		
Bond lengths (Å) [violations]	0.003 [0]	0.003 [0]
Bond angles (°) [violations]	0.547 [0]	0.563 [0]
**Validation**		
MolProbity score	1.38	1.27
MolProbity clashscore	4.16	4.82
Rotamer outliers (%)	0	0
Ramachandran plot		
Overall Z-score [RMSD]	-0.97 [0.35]	-0.68 [0.35]
Favored (%)	96.9	97.9
Allowed (%)	3.1	2.1
Disallowed (%)	0	0
Model-to-data fit		
CC(mask)	0.75	0.75
CC(volume)	0.74	0.72
CC(peaks)	0.45	0.42
EMRinger score	2.33	1.96

© 2020 The Authors.

Appendix Materials and Methods

DNA constructs

Wild-type or truncated UMOD cDNA constructs were cloned in pcDNA3.1(+) (Thermo Fisher Scientific) and HA- or FLAG-tags were inserted after the signal peptide, between T26 and S27 in the protein sequence (Schaeffer *et al*, 2009). Mutation of the hepsin cleavage site (586-RFRS-589/AAAA; 4A mutant) was described previously (Schaeffer *et al*, 2009); constructs pcDNA3.1(+)/UMOD-CCS or pcDNA3.1(+)/UMOD-EHP expressed proteins truncated at residues S589 or S614, respectively. Polymerization interface mutants were generated using a QuikChange Lightning mutagenesis kit (Agilent) following the manufacturer's instructions; primers were designed using the QuikChange Primer Design server (https://www.agilent.com/store/primerDesignProgram.jsp).

Co-expression of FLAG- and HA-tagged UMOD isoforms was performed using a bicistronic vector pVITRO-hygro-mcs (Invivogen). For this purpose, the sequence of FLAG-tagged wt UMOD was subcloned from pcDNA/FLAG-UMOD into the EcoRV site of pVITRO-hygro-mcs, downstream of the mouse elongation factor-1α promoter; the sequences of HA-tagged UMOD isoforms (wt or mutants) were subcloned from pcDNA/HA-UMOD in the Bst1107I site, downstream of the rat elongation factor-1α promoter of the same bicistronic vector.

For mammalian expression of medaka ZI-1,2 (P223-Q591) and ZI-3 (Y74-V420) constructs, synthetic genes (ATUM) were subcloned into vector pHLsec3 (Raj *et al*, 2017), in frame with a sequence encoding a C-terminal 6His-tag.

All DNA constructs were verified by DNA sequencing (Eurofins Genomics) before transfection.

Recombinant protein expression and purification

For characterization of UMOD mutants, stable populations of MDCK cells (ATCC CCL-34) were generated by transfection with Lipofectamine 2000 (Thermo Fisher Scientific) following the manufacturer's protocol. Cells were grown in Dulbecco's Modified Eagle's Medium (DMEM) supplemented with 10% fetal bovine serum, 200 U ml^{-1} penicillin, 200 µg ml^{-1} streptomycin and 2 mM glutamine at 37°C, 5% CO_2; selection was started 24 h after transfection by adding 0.5 mg/ml G418 (Thermo Fisher Scientific) and was pursued for 1-2 weeks in order to obtain a population of G418-resistant cells.

For analyzing the oligomerization state of egg coat protein precursors, medaka ZI-1,2 (which does not contain N-glycosylation sites) was expressed in HEK293T cells (a kind gift of Prof. A.R. Aricescu, University of Oxford, U.K.) (DuBridge *et al*, 1987) grown in DMEM medium supplemented with 4 mM L-Gln, 2% fetal bovine serum and transiently transfected using 25 kDa branched PEI (Aricescu *et al*, 2006; Bokhove *et al*, 2016b), whereas GnTI-deficient HEK293S cells (ATCC CRL-3022) (Reeves *et al*, 2002) were used to express ZI-3 carrying Endoglycosidase H (Endo H)-sensitive Man5GlcNac2 N-glycans. These carbohydrate chains were then enzymatically trimmed to single GlcNAc residues during protein purification, which was performed by batch immobilized metal ion affinity (IMAC) using nickel agarose slurry (Ni-NTA, QIAGEN or Ni Sepharose High Performance, GE Healthcare) and size-exclusion chromatography (SEC) using a Superdex 200 Increase 10/300 GL column (GE Healthcare) equilibrated against 20 mM HEPES pH 7.5, 150 mM NaCl (Bokhove *et al*, 2016b).

 © 2020 The Authors.

Immunoblot

Cell lysis, medium precipitation and western blot experiments were performed essentially as described (Schaeffer *et al*, 2009), using mouse purified anti-HA.11 Epitope Tag monoclonal (clone 16B12) (Biolegend 901502; 1:1,000 dilution), mouse anti-β-actin monoclonal (clone AC-74) (Sigma-Aldrich A2228; 1:20,000 dilution) and rabbit affinity-isolated anti-FLAG polyclonal (Sigma-Aldrich F7425; 1:1,000 dilution).

Immunofluorescence

Cells grown on coverslips were fixed in 4% paraformaldehyde for 15 min, permeabilised for 10 min with 0.5% Triton when indicated and blocked for 30 min with 10% donkey serum. Cells were labelled for 1 h 30 min with the indicated primary antibodies at room temperature, followed by 1 h incubation with the appropriate Alexa-Fluor conjugated secondary antibodies (Thermo Fisher Scientific; 1:500). They were then stained for 5 min with 4,6-diamidino-2-phenylindole (DAPI) and mounted using fluorescence mounting medium (DAKO, Agilent). Pictures were taken with an UltraVIEW ERS spinning disk confocal microscope (Zeiss 63X/1.4, UltraVIEW ERS-Imaging Suite Software; PerkinElmer) or a DM 5000B fluorescence upright microscope (Leica DFC480 camera, Leica DFC Twain Software; Leica Microsystems). All images were imported in Photoshop CS (Adobe) and adjusted for brightness and contrast.

The antibodies used for these experiments were mouse purified anti-HA.11 Epitope Tag monoclonal (clone 16B12) (Biolegend 901502; 1:500 dilution), rabbit affinity-isolated anti-FLAG polyclonal (Sigma-Aldrich F7425; 1:500 dilution), rat anti-HA High Affinity monoclonal (clone 3F10) (Roche 11867423001; 1:500 dilution), goat affinity purified anti-c-Myc polyclonal (Novus Biologicals NB 600-335; 1:500 dilution) and mouse anti-KDEL monoclonal (clone 10C3) (Enzo ADI-SPA-827; 1:200 dilution).

© 2020 The Authors.

Appendix References

Aricescu AR, Lu W, Jones EY (2006) A time- and cost-efficient system for high-level protein production in mammalian cells. *Acta Crystallogr D Biol Crystallogr* 62**:** 1243–1250

DuBridge RB, Tang P, Hsia HC, Leong PM, Miller JH, Calos MP (1987) Analysis of mutation in human cells by using an Epstein-Barr virus shuttle system. *Mol Cell Biol* 7**:** 379–387

Reeves PJ, Callewaert N, Contreras R, Khorana HG (2002) Structure and function in rhodopsin: high-level expression of rhodopsin with restricted and homogeneous N-glycosylation by a tetracycline-inducible N-acetylglucosaminyltransferase I-negative HEK293S stable mammalian cell line. *Proc Natl Acad Sci USA* 99**:** 13419–13424

 © 2020 The Authors.
