# Supplementary material for: Cryo‐EM structure of native human uromodulin, a zona pellucida module polymer
Source: EMBO J. 2020 Nov 16;39(24):e106807. doi: 10.15252/embj.2020106807 (PMC7737619; doi:10.15252/embj.2020106807)
Supplement: Supplementary file 2 — Expanded View Figures PDF [file EMBJ-39-e106807-s002.pdf]

Expanded View Figures

Figure EV1. Full-length UMOD filaments: comparison with elastase-treated material and stability in 6 M urea.

A Domain organization of the secreted human UMOD precursor. Magenta, EGF I-III; salmon, D8C domain; orange, EGF IV; light blue and dark blue, ZP-N and ZP-C domains; red, ZP-N/ZP-C linker; gray, internal hydrophobic patch (IHP); black, CCS; yellow, EHP. A thick black horizontal line marks the CTP, with a brown circle depicting the GPI anchor attachment. Inverted tripods show N-glycans, with the high-mannose chain attached to D8C N275 colored cyan. Black and orange arrows indicate the position of the hepsin (F587|R588) and elastase (S291|S292) cleavage sites, respectively, with thin horizontal bars indicating the extent of $UMOD_{fl}$ and $UMOD_{e}$.

B Representative Volta phase plate micrographs of native $UMOD_{fl}$ filaments. Although tree/front views are predominant, a number of zig-zag/side views can be seen in the right-most micrograph. The yellow arrows show examples of how twisting of individual UMOD filaments generates both views. Scale bars: 50 nm.

C Reducing Coomassie-stained SDS–PAGE analysis of the $UMOD_{fl}$ (6 μg; lane 1) and $UMOD_{e}$ (3 and 5 μg; lanes 2, 3) material used for structure determination.

D Representative micrograph of $UMOD_{e}$ filaments, showing the absence of branches. Scale bar: 50 nm.

E Superposition of the $UMOD_{fl}$ (salmon) and $UMOD_{e}$ (cyan) cryo-EM maps shows that only the former shows density for a globular domain protruding from the core of the filaments. This reveals the approximate location of the elastase cleavage site, corresponding to the N-terminus of $UMOD_{e}$, within the structure of $UMOD_{fl}$ (orange arrows).

F Coomassie-stained SDS–PAGE analysis of supernatant and pellet fractions of purified native UMOD filaments, incubated with increasing amounts of urea. No significant breakdown of the polymers is observed at urea concentrations below 7 M.

Source data are available online for this figure.

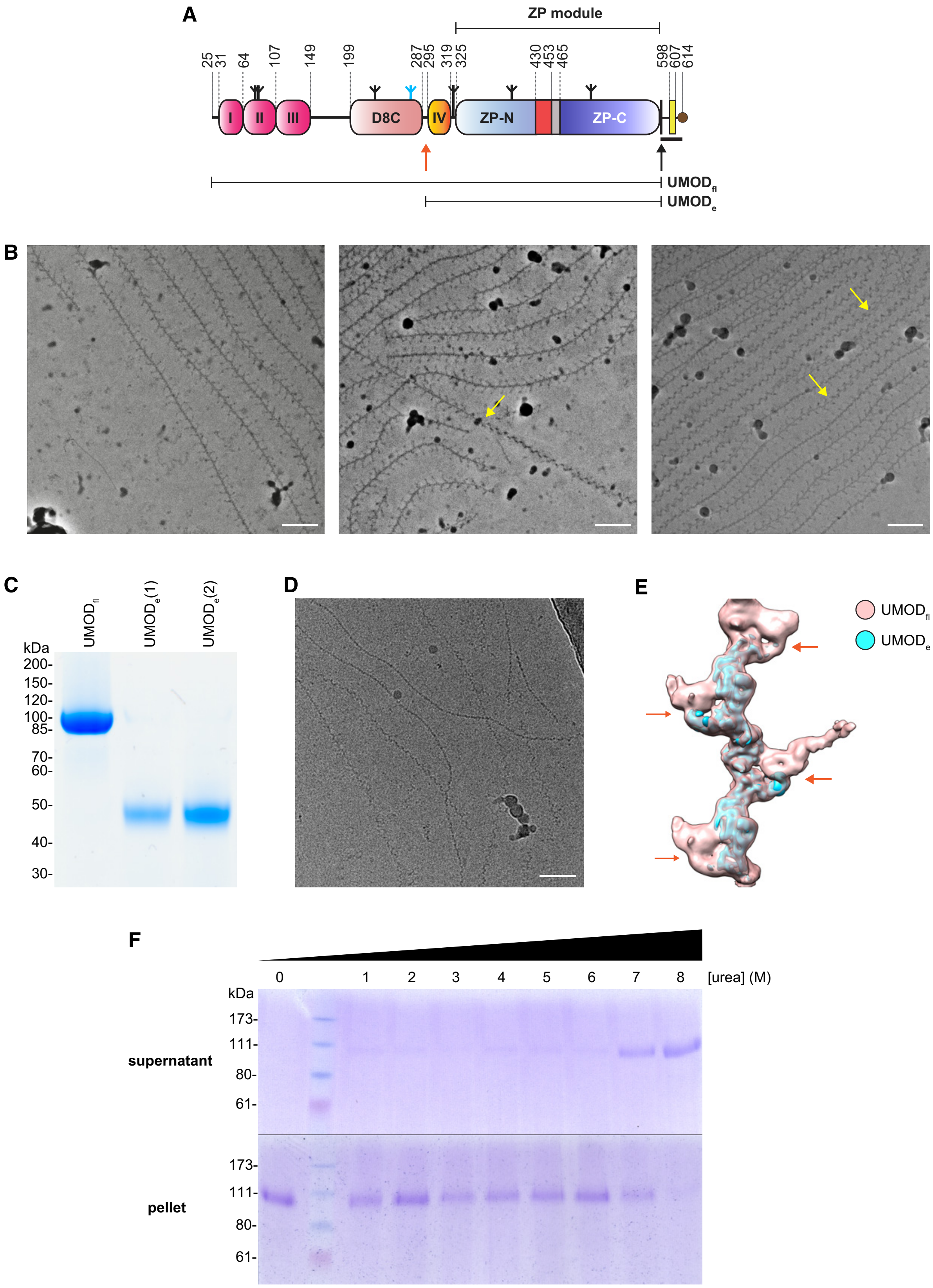

Figure EV1.

 © 2020 The Authors

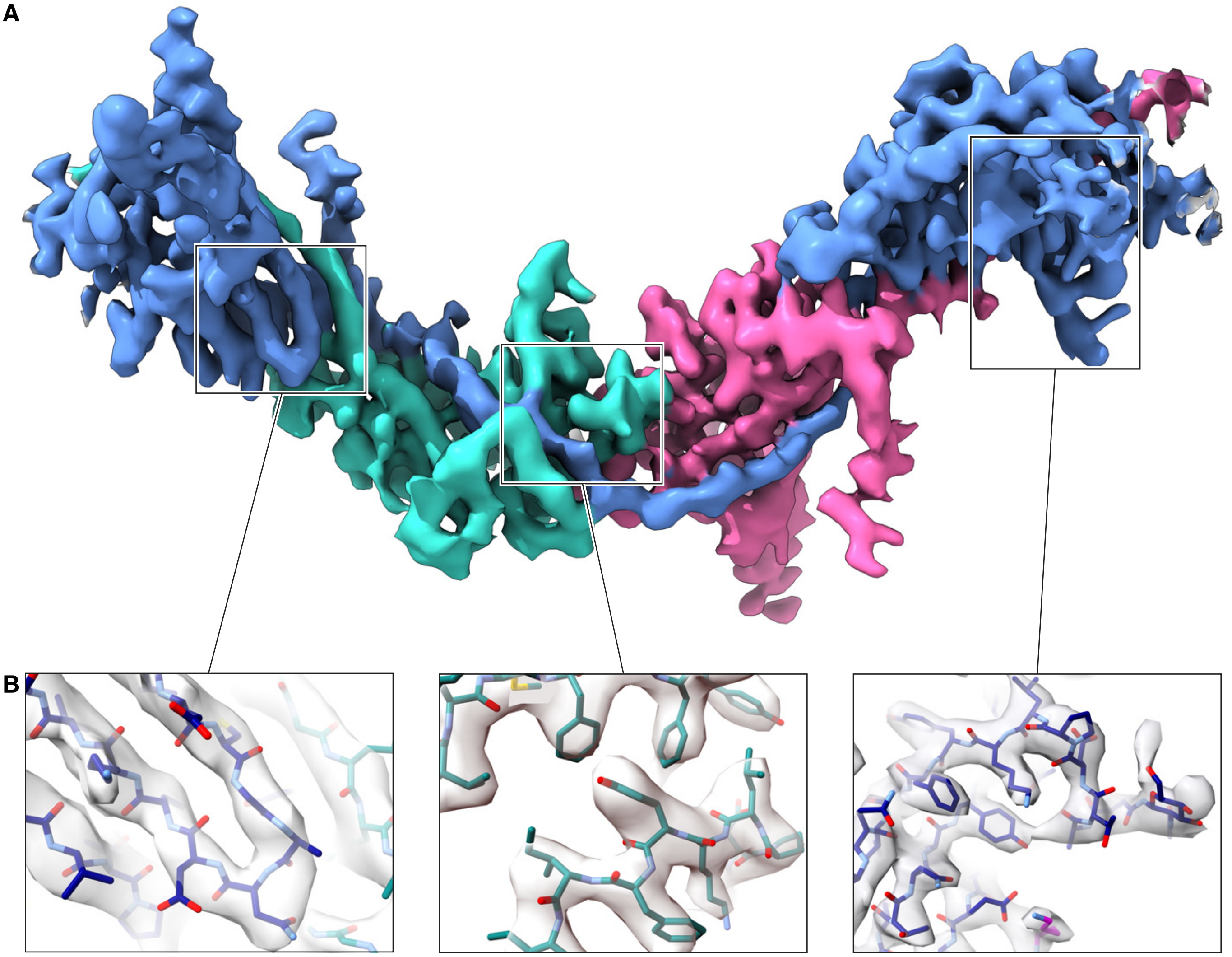

Figure EV2. Sharpened cryo-EM map of the UMOD$_{fl}$ filament core (3.4 Å resolution).

A Overall view of the entire polymerization region of a UMOD molecule (blue), wrapped around the ZP-C domain (teal) and EGF IV + ZP-N domains (magenta) of the preceding and following subunits, respectively.

B Details of different parts of the map, highlighting the separation of β-strands (left panel) and the quality of side chain density (middle and right panels). The map is fitted with an atomic model of UMOD where carbon atoms of different chains are colored according to panel (A).

© 2020 The Authors

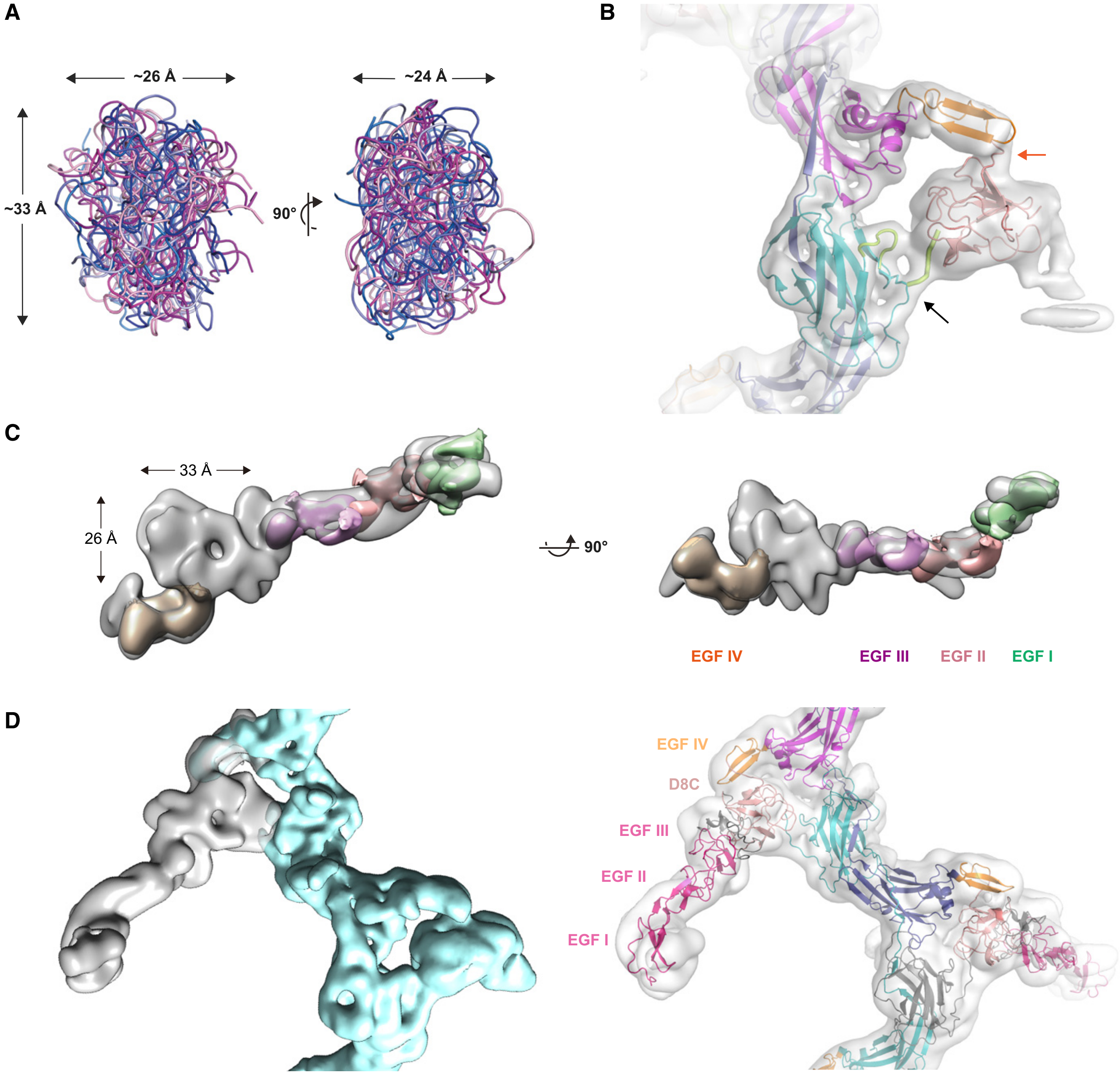

Figure EV3. Docking of D8C and EGF I-III domain models into the density map of $UMOD_{fl}$.

A D8C domain models created using I-TASSER (blue shades) or Robetta (magenta shades) have approximately the same overall dimensions.

B Consistent with the location of the elastase cleavage site (orange arrow) that immediately precedes the EGF IV domain (orange), the top model of D8C generated by Robetta (salmon) can be straightforwardly docked into the globular density protruding from the core of the filament. The unsharpened cryo-EM map of $UMOD_{fl}$ is shown, and a black arrow indicates the C_6527-C_8582 disulfide, which orients the C-terminal tail of mature UMOD (thick lemon tube) toward D8C. The latter also packs against the loop that connects C_6527 to βD (thin lemon tube).

C Using multi-body refinement in RELION, we performed a focused refinement of the density corresponding to the complete N-terminal branch of UMOD. This locally refined map suggested the location of EGF I-III, which served as a guideline to build a model of full-length UMOD.

D The gray density depicts the N-terminal branch, treated as body 1, whereas the cyan density shows the rest of the filament segment, treated as body 2. After being separately refined, these two local maps were merged to produce a composite map of $UMOD_{fl}$, which was used as a reference for model building and as a starting point for Fig 6B. UMOD branch domains are indicated and colored as in Fig EV1A.

 © 2020 The Authors

Figure EV4. UMOD subunit interactions: evolutionary conservation, comparison with the homodimeric cZP3 precursor and consistency with features of other ZP module proteins.

A Surface representation of a UMOD subunit (UMOD 3), with residues colored from green to violet by increasing conservation according to ConSurf. Other subunits within the filament are shown as cartoons colored according to Fig 2, with interface areas highlighted in panels (B-D) indicated by red rectangles.

B Interface between the interdomain linker of a UMOD subunit and the ZP-C domain of the subunit that precedes it. White labels in this panel as well as panels (C and D) indicate residues depicted in surface representation.

C Close-up of the interface between the ZP-C domain of a UMOD molecule and the ZP-N E′FG extension of the subunit that follows it.

D Details of the ZP-C αEFβ/ZP-N βF′ interface involving a copy of UMOD (UMOD n) and the second to next subunit (UMOD n + 2).

E Superposition of the ZP-C domains of a polymeric UMOD subunit (UMOD 2 of Fig 2; teal) and one of the two subunits of the cZP3 precursor (chain A of PDB 3NK3; yellow, with the ZP3-specific subdomain colored hot pink). The ZP-N counterparts (magenta and orange-yellow, respectively) that interact with these domains are differently positioned relative to the corresponding ZP-Cs, although both interfaces are formed by the same elements. The arrow indicates how superimposing the ZP-C domains brings the EHP of ZP3 in the same position as α1β in the interdomain linker of the UMOD subunit that follows UMOD 2 (UMOD 3; blue).

F Same view of the ZP-C αEFβ/ZP-N βF′ interface shown on the right half of panel (D), with the ZP-C surface colored by calculated electrostatic potential from −5 kT/e (red) to + 5 kT/e (blue) through 0 kT/e (white).

G The ZP module interface observed in the UMOD filament is compatible with the expected solvent exposure of the N- and O-glycosylation sites of other ZP module proteins. Predicted N-glycosylation sites of human glycoprotein 2 (orange), α- and β-tectorin (yellow and blue), ZP2 (green), ZP3 (gray), ZP4 (cyan), chicken ZPD (purple), as well as O-glycosylation site 1 of chicken ZP3 (black), are mapped onto the surface of three adjacent UMOD chains (A, light blue; B, light teal; C, light magenta) based on sequence–structure alignments. UMOD N-glycosylation sites are shown in red.

H Homology models of the N-terminal repeat region (NTR) of mouse ZP2 plus its ZP-N domain (Monné *et al*, 2008) and the ZP-C domain of mouse ZP3 (green) were superimposed on the ZP-N and ZP-C domains of two adjacent UMOD filament subunits (gray), respectively; subsequently, ZP2 NTR was approximately oriented like the N-terminal branch of the same UMOD subunit used for the ZP-N/ZP-N superposition. The resulting model shows that, akin to UMOD EGF I-III+D8C, the ZP-N domain repeats that precede the ZP module of ZP2 can project from the core of egg coat filaments without interfering with subunit polymerization interfaces. Similarly, the C-terminal subdomain specific to ZP3 (hot pink) is predicted to be positioned laterally to the egg coat filament body and potentially face the ovastacin cleavage site in the second N-terminal repeat of ZP2 (spheres).

© 2020 The Authors

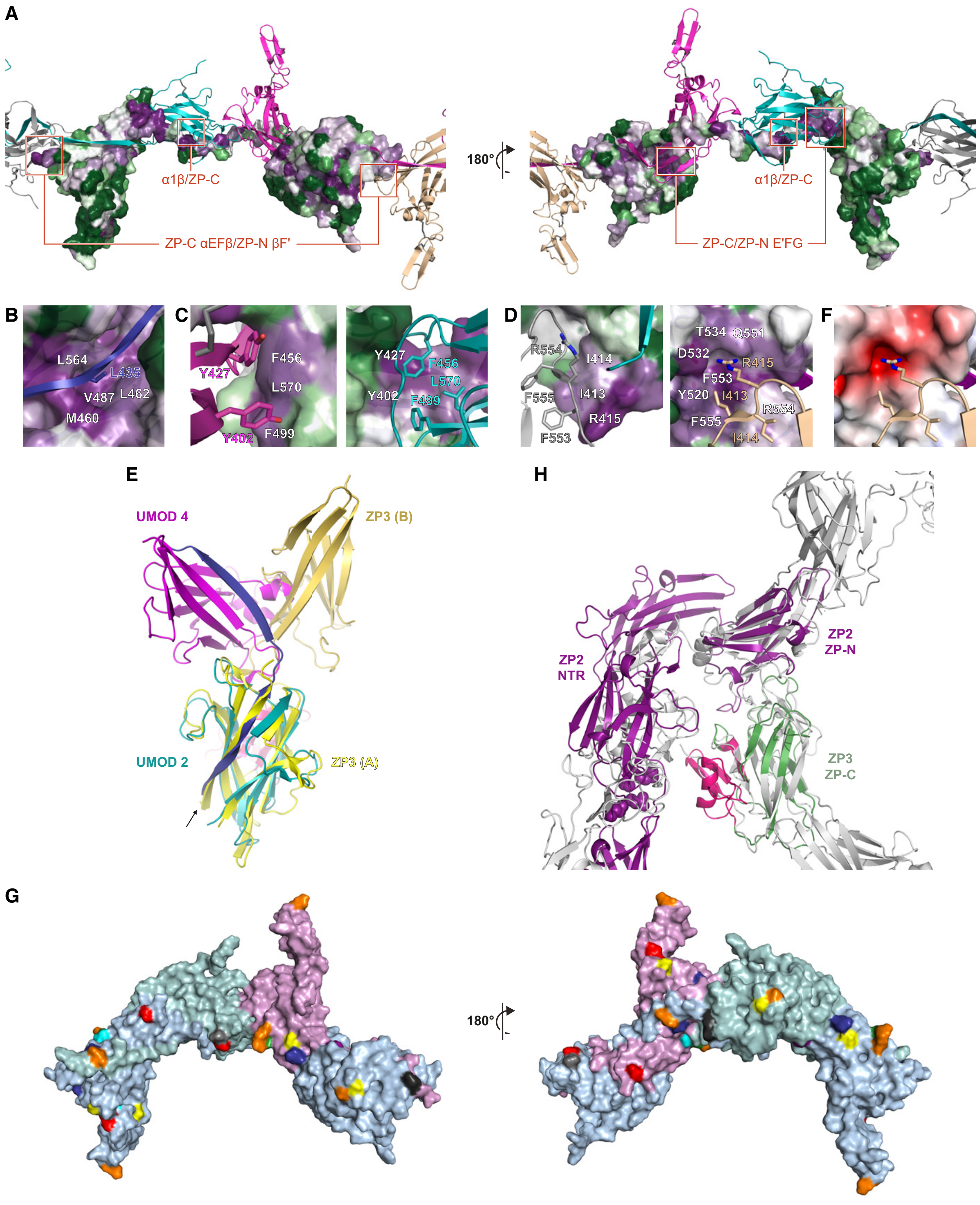

Figure EV4.

 © 2020 The Authors

Figure EV5. Analysis of the effects of UMOD mutations on protein expression, secretion and filament formation.

A Immunofluorescence of permeabilized MDCK cells expressing full-length wt UMOD or mutants R415A or ΔFA. No intracellular aggregation is observed. Scale bar: 50 μm.

B MDCK cells stably expressing R415A or ΔFA mutants of full-length UMOD. Immunoblots of total cell lysates (top panels) indicate that both wt protein and polymerization mutants are mainly present as a fully glycosylated isoform (upper band, black arrow), in addition to a minor ER-glycosylated species (lower band, gray arrow). Immunoblot analysis of PNGase F-deglycosylated proteins secreted by MDCK cells (bottom panels) shows that neither the R415A nor the ΔFA mutation affects protein secretion in the culturing medium. Blue arrows indicate proteins cleaved within the juxtamembrane region between GPI and EHP; red arrows mark proteins that were processed at the CCS. These results demonstrate comparable intracellular trafficking and secretion of wt and mutant isoforms. Note that the ΔFA mutation increases the amount of protein that is processed at the CCS, suggesting that alteration of the FXF motif affects the accessibility of the closely located cleavage site (Fig 3B, left panel); despite this, filaments are completely absent in the case of the mutant (Fig 4A, right panel), further underlying the specific effect of the mutation on UMOD polymerization.

C Immunoblot of deglycosylated proteins released in the culturing medium of MDCK cells stably co-expressing FLAG-tagged wt UMOD and HA-tagged wt or mutant isoforms. Co-expression of mutant UMOD does not alter the cleavage of the FLAG-tagged wt protein, suggesting that the dominant-negative effect of the ΔFA and 4A mutants is not caused by abnormal processing of wt UMOD.

D Immunoblot of UMOD in the cell lysate and conditioned medium of MDCK cells transiently transfected with the indicated HA-tagged isoforms. The presence of the EHP motif is required for efficient protein exit from the ER, as suggested by comparing the intracellular levels and secretion of UMOD-CCS and UMOD-EHP.

E MDCK cells transiently expressing ZP-N R415A and ZP-C ΔFA mutants of UMOD-EHP. Immunofluorescence of permeabilized cells shows the absence of intracellular polymers in both wt and mutant forms. Scale bar: 10 μm.

F Immunofluorescence analysis of unpermeabilized MDCK cells expressing UMOD-EHP. Lack of membrane-anchoring prevents localization and polymerization of the protein at the plasma membrane. Scale bar: 50 μm.

G Co-culture of MDCK cells stably expressing HA-tagged (red) or Myc-tagged (green) wt UMOD. Filaments are uniformly colored, suggesting that polymerization depends on incorporation of membrane-bound monomers instead of cleaved monomers released in the culture medium. Scale bar: 25 μm.

Source data are available online for this figure.

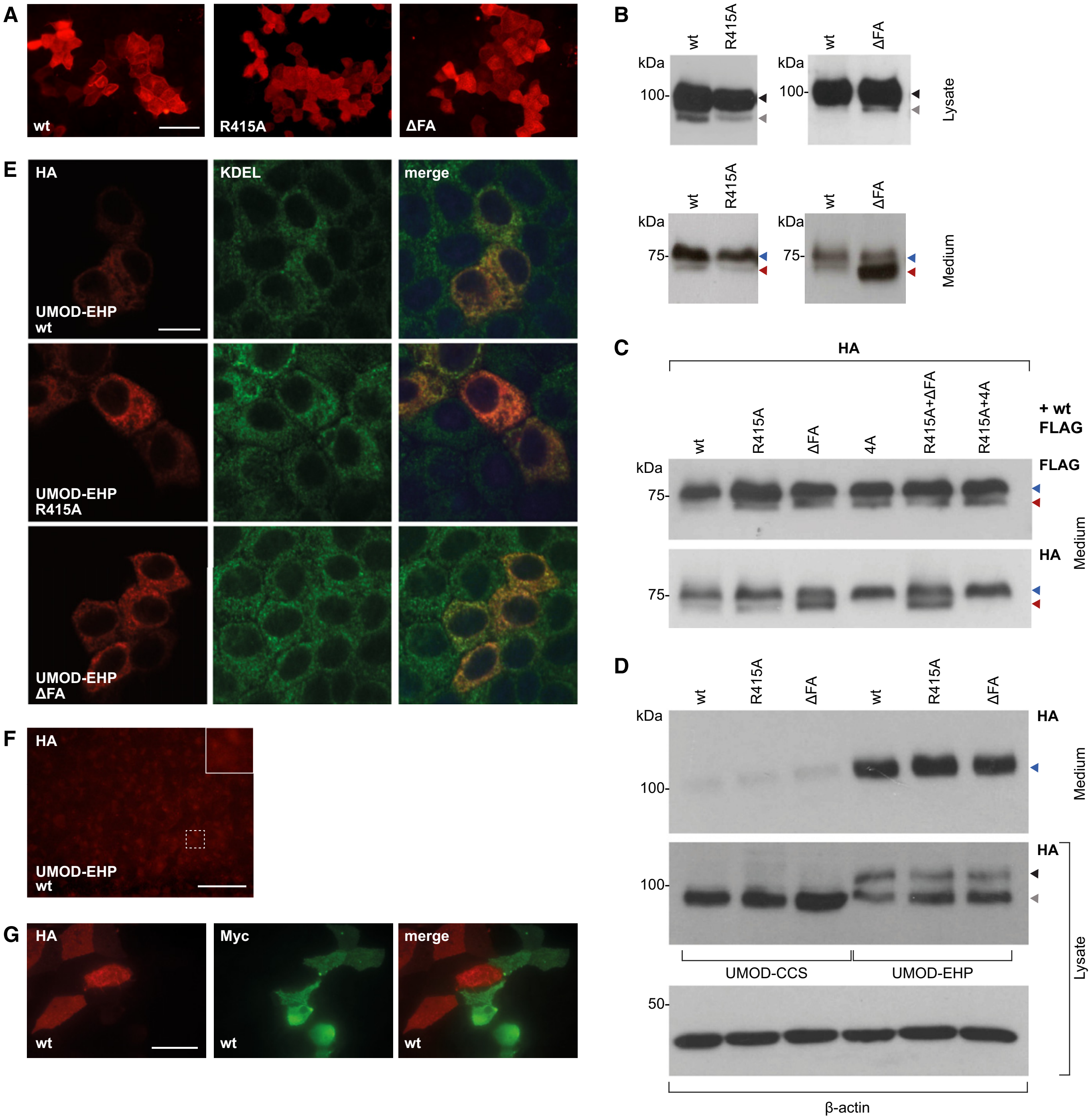

Figure EV5.

 © 2020 The Authors
