# Supplementary material for: Cryo‐EM structure of native human uromodulin, a zona pellucida module polymer
Source: EMBO J. 2020 Nov 16;39(24):e106807. doi: 10.15252/embj.2020106807 (PMC7737619; doi:10.15252/embj.2020106807)
Supplement: Supplementary file 3 — Movie EV1 [file EMBJ-39-e106807-s003.zip › EMBOJ-2020-106807R_MovieEV1/EMBOJ-2020-106807R_MovieEV1.docx]

**Movie EV1. Details of the sharpened cryo-EM map of UMOD_fl_ at 3.8 Å resolution (I).**

Interaction between the glycan attached to N396 of the ZP-N domain of chain C (UMOD 4) and β1 R449 in the interdomain linker of chain A (UMOD 3).
