# Supplementary material for: Cryo‐EM structure of native human uromodulin, a zona pellucida module polymer
Source: EMBO J. 2020 Nov 16;39(24):e106807. doi: 10.15252/embj.2020106807 (PMC7737619; doi:10.15252/embj.2020106807)
Supplement: Supplementary file 4 — Movie EV2 [file EMBJ-39-e106807-s004.zip › EMBOJ-2020-106807R_MovieEV2/EMBOJ-2020-106807R_MovieEV2.docx]

**Movie EV2. Details of the sharpened cryo-EM map of UMOD_fl_ at 3.8 Å resolution (II).**

Map region around the α1β strand formed by the interdomain linker of chain A (UMOD 3).
