# Supplementary material for: Cryo‐EM structure of native human uromodulin, a zona pellucida module polymer
Source: EMBO J. 2020 Nov 16;39(24):e106807. doi: 10.15252/embj.2020106807 (PMC7737619; doi:10.15252/embj.2020106807)
Supplement: Supplementary file 6 — Movie EV4 [file EMBJ-39-e106807-s006.zip › EMBOJ-2020-106807R_MovieEV4/EMBOJ-2020-106807R_MovieEV4.docx]

**Movie EV4. Details of the sharpened cryo-EM map of UMOD_fl_ at 3.8 Å resolution (IV).**

Interface between αEFβ of chain B (UMOD 2) and βF' of chain C (UMOD 4).
