# Supplementary material for: Cryo‐EM structure of native human uromodulin, a zona pellucida module polymer
Source: EMBO J. 2020 Nov 16;39(24):e106807. doi: 10.15252/embj.2020106807 (PMC7737619; doi:10.15252/embj.2020106807)
Supplement: Supplementary file 7 — Movie EV5 [file EMBJ-39-e106807-s007.zip › EMBOJ-2020-106807R_MovieEV5/EMBOJ-2020-106807R_MovieEV5.docx]

**Movie EV5. Proposed mechanism of UMOD polymerization.**

UMOD CCS cleavage by hepsin is represented by a flash in the animation.
