# Supplementary material for: Cryo‐EM structure of native human uromodulin, a zona pellucida module polymer
Source: EMBO J. 2020 Nov 16;39(24):e106807. doi: 10.15252/embj.2020106807 (PMC7737619; doi:10.15252/embj.2020106807)

Source data

Fig EV1C

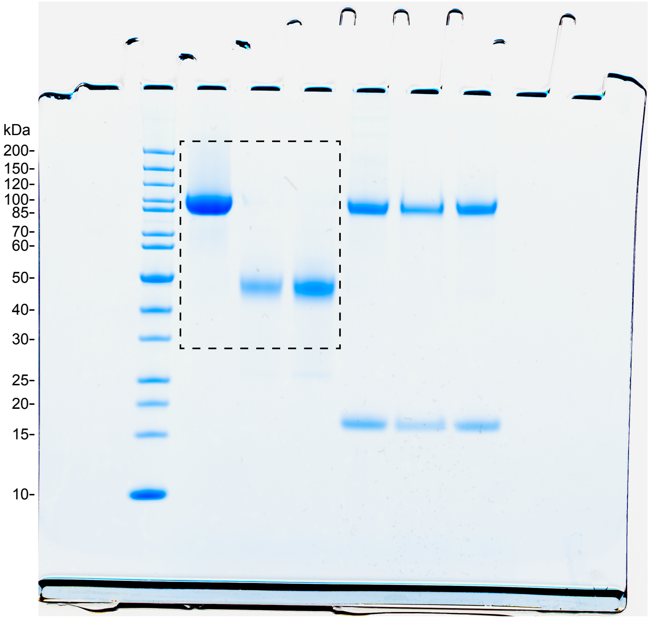

Fig EV1F

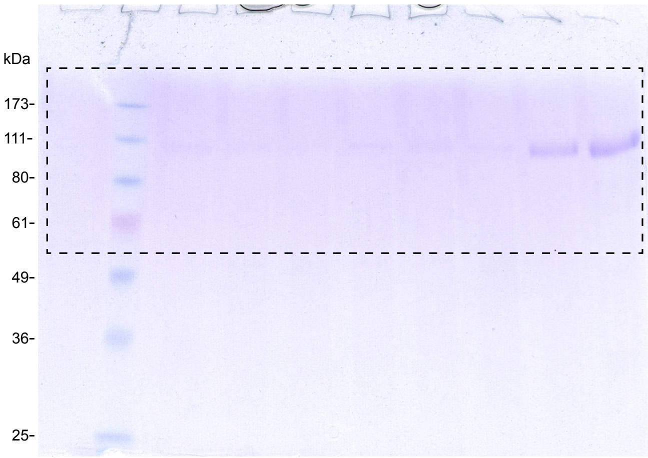

Fig EV1F

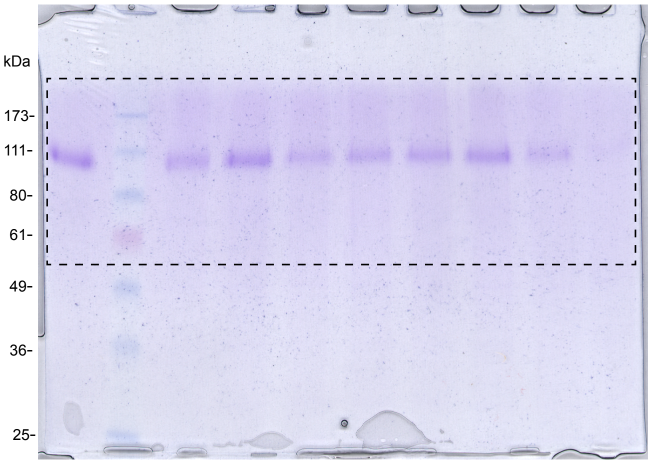

Fig EV5B

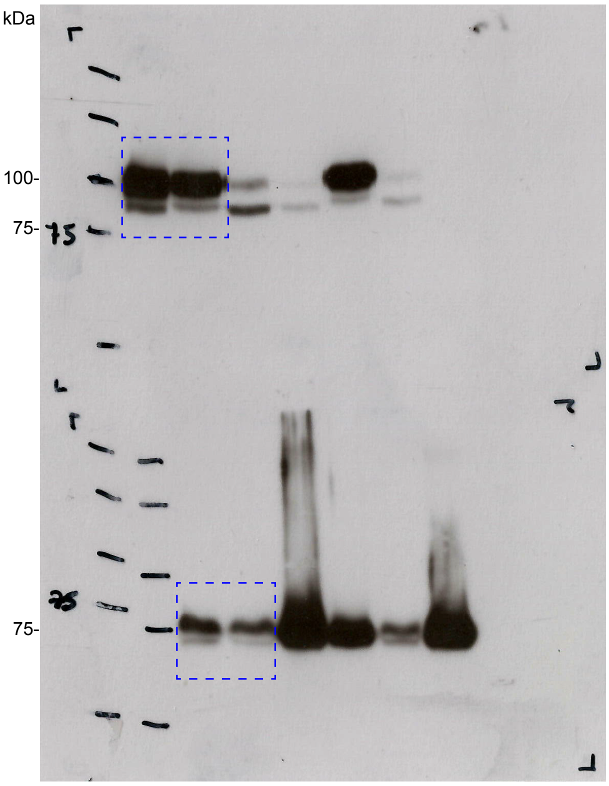

Fig EV5B

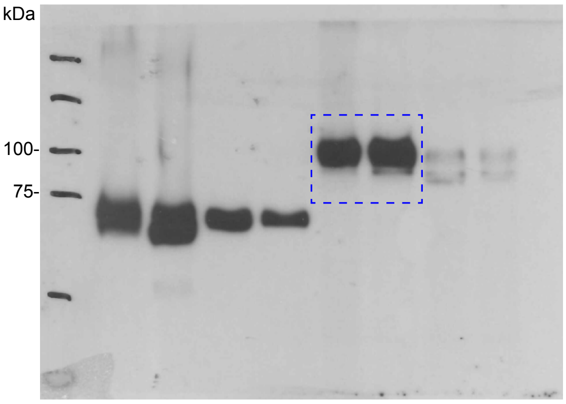

Fig EV5B

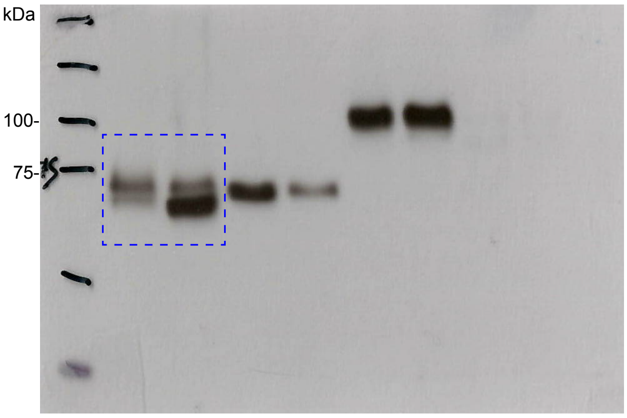

Fig EV5C

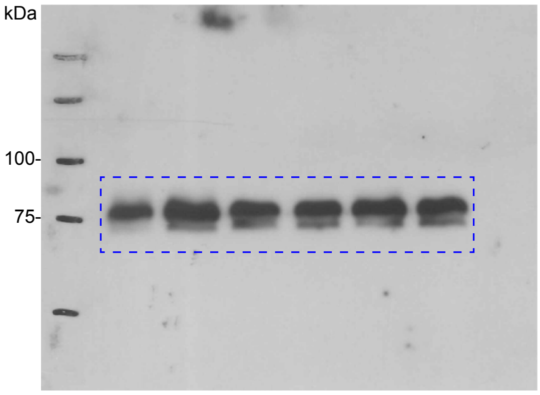

Fig EV5C

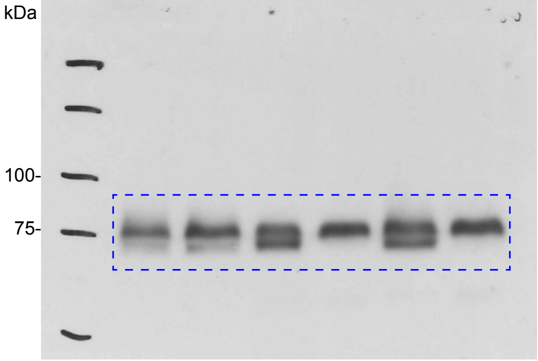

Fig EV5D

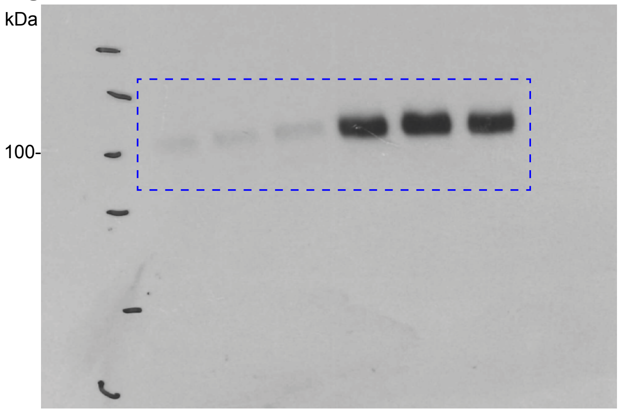

Fig EV5D

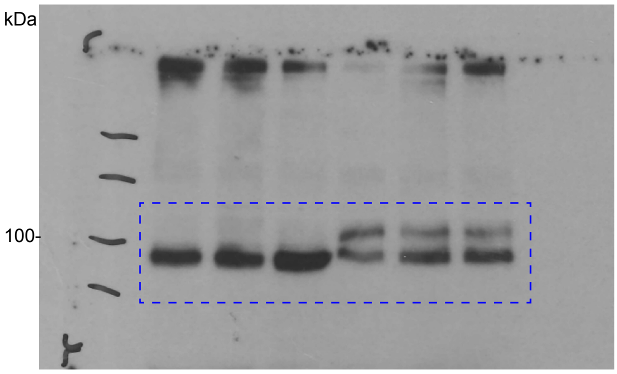

Appendix Fig S3A

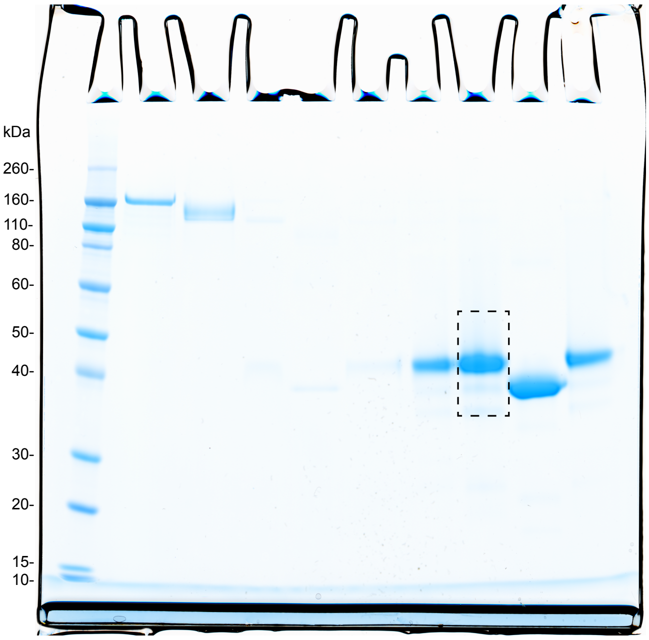

Appendix Fig S3B

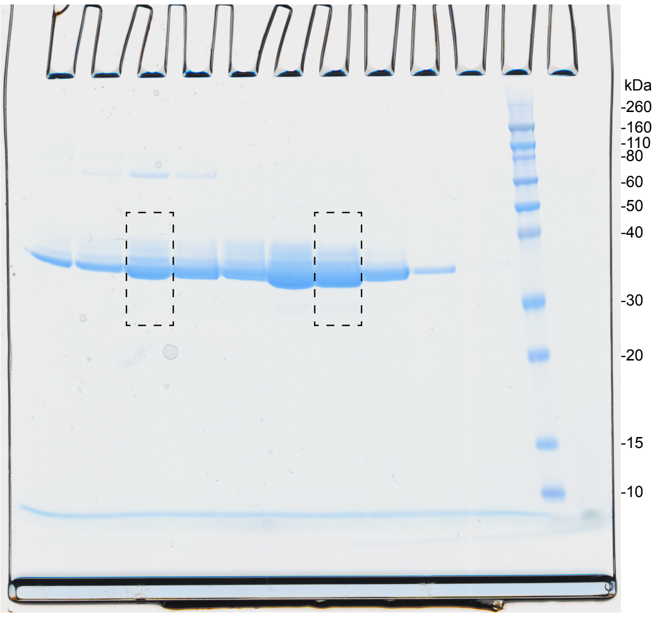

Fig EV5D

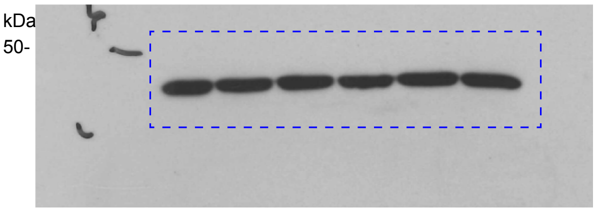

Supplement: Supplementary file 8 — Source Data for Expanded View/Appendix [file EMBJ-39-e106807-s008.pdf]
